# Distinct mechanisms of the inhibition of vasculogenesis by different species of ionizing particles

**DOI:** 10.1093/jrr/rrt172

**Published:** 2014-03

**Authors:** Peter Grabham, Preety Sharma, Alan Bigelow, Charles Geard

**Affiliations:** 1 Center for Radiological Research, Columbia University, VC 11-205A/243, 630 West 168th Street, New York, NY 10032, USA; 2 Radiological Research Accelerator Facility, Center for Radiological Research, Nevis Laboratory, Columbia University, 136 S. Broadway, Irvington, NY 10533, USA

**Keywords:** angiogenesis, vasculogenesis, radiation, charged particles

## Abstract

The human vasculature includes a vast network of microcapillaries networking the body and is a major target for non-carcinogenic effects of space radiation in the body. The brain microvascular system is crucial to healthy functioning of the brain and its pathology is not only a primary event in a range of neurodegenerative diseases but also an important influencing factor in many others. The vasculature is maintained by angiogenesis regenerating vessels as they are needed, this is particularly relevant if the blood–brain barrier is damaged by agents such as space radiation, thereby creating the need for angiogenic regeneration. The resulting lack of vasculature due to the inhibition of re-growth of vessels can, in turn, lead to a negative feedback loop and further pathologies.

Using three-dimensional human vessel cultures with human umbilical vein and brain microvascular endothelial cells, we have developed assays that determine at what stage angiogenesis is inhibited by ionizing radiation. The relative biological effect of high linear energy transfer (LET) 1 GeV Fe ions compared with low LET 1 GeV protons is only one for developing vessels but greater than four for mature vessels. This action of low LET protons on developing vessels was surprisingly effective (50% inhibition with 40 cGy exposure) and together with the effect of high LET ions may represent a significant hazard in the space environment.

The morphology of developing vessels 5 days after exposure showed significant differences that suggest distinct mechanisms of inhibition. Cells exposed to protons failed to make connections with other cells. Conversely, cells exposed to Fe ions extended cellular processes and made connections to other cells but did not develop a central lumen. The microtubule and actin cytoskeletons showed differences indicating that motility at the extending tips of endothelial cells is inhibited by protons but not Fe ions. Actin-rich protrusive structures that contain bundled and dynamic microtubules showed a 65% decrease when exposed to high-energy protons but not with the same dose of high-energy Fe ions. Since protein kinase C (PKC) has long been known to stimulate angiogenesis, we hypothesized that rescue of the capillary phenotype after proton exposure would be possible by stimulating PKC before irradiation. One-day-old vessel cultures were treated with 30 and 60 nM phorbol ester (PMA) 15 min before irradiation. Stimulation of PKC restored capillary formation in proton-treated cultures but not in Fe ion-treated cultures. More specifically, stimulation of PKC by PMA was able to restore the tip motility that was inhibited by low LET ions [ 1].

Further studies with various charged particles showed that low LET ion particles (Proton and He ions) with an LET lower or equal to 1 keV/μm inhibit vasculogenesis in the same way as protons. Higher LET charged particles (Silicon 1GeV, Oxygen 250 MeV and 1 GeV and Carbon 290 MeV and 1 GeV) with an LET ≥8 keV/μm inhibit vasculogenesis in the same way as Fe ions.

In conclusion, we have shown that low and high LET ions inhibit the formation of brain capillaries by different mechanisms. For low LET ions, inhibition involves regulation of PKC-dependent motile tips leading to a failure of cellular processes to migrate through the matrix and meet up with other processes. For high LET ions, the cells fail to complete angiogenesis by not migrating and forming tubular structures. This complexity of response opens up possibilities of greater control over angiogenesis and the resulting pathologies during coincident exposure or therapy. For exposure in space, knowledge of these mechanisms will enable more precise risk assessment and mitigation strategies. For radiotherapy, treatment could be manipulated to utilize the radiation effectively.
